# ASSOCIATION BETWEEN DUAL TASK FUNCTION AND NEUROPSYCHOLOGICAL TESTING IN OLDER ADULTS WITH COGNITIVE IMPAIRMENT

**DOI:** 10.1093/geroni/igac059.2315

**Published:** 2022-12-20

**Authors:** Kelsi Petrillo, Nima Toosizadeh

**Affiliations:** University of Arizona, Tucson, Arizona, United States; University of Arizona, Tucson, Arizona, United States

## Abstract

Cognitive impairment is an increasingly relevant health concern, as demonstrated through data estimates that by 2040 the number of older adults with dementia will surpass nine million in the United States. Despite this high prevalence, more than half of patients with dementia never receive an evaluation. This indicates that a quick and objective routine test for screening cognitive decline in older adults is needed. Poor dual-task gait performance has been associated with decreased executive and neuropsychological function. However, gait tests are not always viable for clinics or older patients. The aim of this study was to assess the relationship between a novel upper-extremity function (UEF) dual-task performance and neuropsychological test results in older adults. We recruited older adults at three stages: cognitively normal (n=33), mild cognitively impaired (n=34), and Alzheimer’s disease (n=22). For UEF tasks, participants performed a consistent elbow flexion, while counting backwards in by threes. Wearable motion sensors were attached to forearm and upper-arm to measure accuracy and speed of elbow flexion kinematics and the UEF cognitive score. The results demonstrate a strong correlation between UEF cognitive score and MMSE, Mini-Cog, Category fluency, Benson complex figure copy, Trail making test, and MOCA (r values between -0.2355 and -0.6037 and p< 0.0288). UEF dual-task was associated with executive function, orientation, repetition, abstraction, verbal recall, attention and calculation, language and visual construction. The results from this study convey a promising future for the use of dual task UEF as a safe and convenient cognitive impairment screening.

